# How Do Patients Understand Safety for Cardiac Implantable Devices? Importance of Postintervention Education

**DOI:** 10.1155/2018/5689353

**Published:** 2018-06-24

**Authors:** Bekir Serhat Yildiz, Gulin Findikoglu, Yusuf Izzettin Alihanoglu, Ismail Dogu Kilic, Harun Evrengul, Hande Senol

**Affiliations:** ^1^Department of Cardiology, Faculty of Medicine, University of Pamukkale, Denizli, Turkey; ^2^Department of Physical Medicine and Rehabilitation, Faculty of Medicine, University of Pamukkale, Denizli, Turkey; ^3^Department of Biostatistics, Faculty of Medicine, University of Pamukkale, Denizli, Turkey

## Abstract

**Aim:**

This study was designed to assess the effect of patient education on the knowledge of safety and awareness about living with cardiac implantable electronic devices (CIEDs) within the context of phase I cardiac rehabilitation.

**Methods:**

The study was conducted with 28 newly implanted CIED patients who were included in “education group (EG)”. Patients were questioned with a survey about living with CIEDs and electromagnetic interference (EMI) before and 1 month after an extensive constructed interview. Ninety-three patients who had been living with CIEDs were included in the “without education group (woEG)”.

**Results:**

Patients in EG had improved awareness on topics related to physical and daily life activities including work, driving, sports and sexual activities, EMI of household items, harmful equipment, and some of the medical devices in the hospital setting (p<0.05). Patients in EG gave significantly different percent of correct answers for doing exercise or sports, using the arm on the side of CIEDs, EMI of some of the household appliances, medical devices, and all of the harmful equipment compared to woEG (p<0.05).

**Conclusion:**

It was demonstrated that a constructed education interview on safety of CIEDs and living with these devices within the context of phase I cardiac rehabilitation is important for improving the awareness of patients significantly. Thus, patients might achieve a faster adaptation to daily life and decrease disinformation and misperceptions and thus promote the quality of life after the device implantation.

## 1. Introduction

Currently, millions of people with cardiac implantable electronic devices (CIED) are living with us and hundreds of thousands join them every year [1, 2]. Implantable cardioverter-defibrillators (ICD) have saved lives of patients at risk of sudden death due to ventricular arrhythmias. Pacemakers (PM) have improved symptoms in patients with bradyarrhythmias and cardiac resynchronization devices have decreased mortality and morbidity and increased quality of life for patients with heart failure [3].

Extrinsic factors such as trauma that damages the device, electromagnetic radiation, and lead displacement may cause ICDs and pacemakers to provide insufficient or incorrect therapy. These complications are called “induced device malfunction” that necessitate device removal often [4]. Moreover, the increase in survival time, the younger age of recipient patients, and the increase in the complexity of device and procedure have increased the risk of failures related to components of the system [5]. Most researchers indicated that well-planned education programs could help patients to avoid more intense treatment in the time following implantation [6–8].

Although the CIEDS do not have an adverse effect on lifestyle, they evoke concerns and anxiety related to the activities and daily lives of the patients. This may in part result from myths, misconceptions, and disinformation [9, 10] provided most of the time by nonprofessional people in the patient's social life like family, relatives, neighbors, or others [9, 11]. If recipients of CIEDs do not receive information following implantation, patients may develop uncertainty about the future, restrict their activities, or develop psychological problems [12]. Actually, many studies have consistently shown that patient education materials are not understood by most adults [13]. A few small studies with psychoeducational intervention have demonstrated improvements in anxiety, depression, quality of life, and physical outcomes. Furthermore, fewer unplanned hospital admissions and calls to healthcare providers were noted [14, 15]. Studies have shown that ICD-implanted patients have a higher demand for information regarding the device and living with it [6, 16].

Rehabilitation of patients with CIEDs comprises secondary prevention of underlying cardiac disease, training activities, psychological support, and informative education programmes [17]. Patient-centered care and constructed educational programme within the context of phase I cardiac rehabilitation could decrease induced device malfunction and help clinicians to attend to the physical and emotional needs of the patients. Thus, patients will be able to deal with misconceptions more efficiently to regain a high quality of life.

Although the effect of psychological intervention on the level of anxiety and depression was investigated in the literature, little is known about the effect of patient education program. This study was designed to assess the effect of patient education on the knowledge of safety and awareness about living with cardiac implantable electronic devices (CIED) within the context of phase I cardiac rehabilitation.

## 2. Materials and Methods

This study was conducted as a single center prospective, single blinded study in the Cardiology Department of University of Pamukkale Hospital. Bedridden patients or patients with hemiplegia or with a psychiatric diseases were excluded. Patients who were unable to cooperate or who were not willing to participate or who were not permitted by their physician due to the clinical status were not included. Demographic and clinical data were collected from medical records.

Fifty patients all with newly implanted device were recruited from clinic with random order. 40 of them were suitable with respect to inclusion or exclusion criteria. They were consulted for an educational interview in the day following implantation and were included in the “education group (EG)”. Patients responded to a survey measuring the knowledge of the patients about living with CIEDs before interview. Then, an extensive constructed interview was conducted both verbally and visually by projected images to provide a standardized content. Patients and their close relatives were accepted for the interview and allowed to ask questions freely. 50-minute long education programme covered topics on how heart and devices work basically, shapes, physical properties and types of the devices, longevity, replacement and controls of device, wound care, personal hygiene, cautions related to healing period, restrictions on effort and movements made with the arm on the site of implantation, and daily life activities including sport activities, work, sexual life, and driving. Definition of electromagnetic interference (EMI) was introduced; then, EMI related to household appliances, office items, medical devices found in the hospital setting, and harmful equipment with strong electromagnetic fields were explained to patients. Then, they were questioned about any particular devices or activities in their individual life. Patients were followed for 1 month and asked to fill the survey again. All of the patients were called for control. 28 patients attended 1 month after device implantation. Patients who did not come at time were not included. Responses before and after education programme were used to compare the effectiveness.

The data of the patients included in the “without education group” (woEG) were withdrawn from our previous study [18]. 120 patients with a permanent CIED had been selected with the help of software from registration documents of the clinic. 93 of them had been found to be eligible with respect to inclusion and exclusion criteria. The age and the sex of the patients were not statistically different from EG. These patients had been briefly informed about living with a CIED following implantation by a cardiologist in the clinic during routine practice or had been living with devices for a long time.

The survey developed making use of literature review, expert opinions, and patient guides from several clinics and manufacturers and it was introduced into the literature by Yildiz et al. [18]. The survey was composed of 36 questions prepared in a self-administered form. A Cronbach alpha value of 0.922 was found for the internal validity of the test [18]. This survey used for this study composed of 4 sets of questions. First set questioned the perceptions of patients with CIEDs of physical activities of daily life. Second set contained questions on the safety of household appliances for EMI. Harmful electrical equipment that has a strong potential to induce CIED malfunction was questioned in the third set. Fourth and the last set was on the medical devices used in the hospital setting that may or may not have a potential to cause EMI. Illiteracy was defined as inability to read or write. Illiterate patients who cannot read or write were assisted for only the questions by a medical stuff or relatives of the patients. Answers were evaluated with respect to North American Society of Pacing and Electrophysiology (NASPE) Practice Guideline, 2001 [19]. Patients were asked to select one of the responses: “correct”, “wrong”, or “no idea”. Responses selected as “wrong” or “no idea” were grouped and considered incorrect.

### 2.1. Ethics

The study conforms to the ethical guidelines of the 1975 Declaration of Helsinki and was approved by Ethics Committee of University of Pamukkale. Written consent was obtained from all subjects who were completely informed about the study.

### 2.2. Statistical Analyses

Continuous data were expressed as mean ± standard deviation and categorical data was expressed as number and percent. McNemar test was used to detect changes within the EG. Chi-Square test was used for the comparison between EG and woEG. All analysis was executed in SPSS 17.0 and p< 0.05 was considered statistically significant.

## 3. Results

Twenty-eight patients out of 40 were included for the final assessment in EG (Table 1). Mean age of the patients was 60.54±10.58 years in EG and 65.41±14.40 in woEG. Male/female ratio was 17/11 (60.7 % / 39.3%) and 59/34 (63.4% / 36.6%) in EG and woEG, respectively. 16 (57.1 %) and 52 (55.91 %) of the patients were graduated from primary or secondary schools and 14 (50 %) and 48 (51.61 %) of the patients were retarded in EG and woEG, respectively.

Indications for implantations were atrioventricular and sinus node dysfunction for 11 (39.28 %) and 37 (39.8 %) versus 8 (28.57 %) and 31 (33.3 %) of the patients in EG and woEG, respectively (Table 2). The 2 very frequent underlying etiologies were cardiomyopathy and ischemic heart disease by 46.4 and 21.4% in EG; however there were ischemic heart disease and degeneration by 32.2 and 30.1% in woEG. The most commonly used type or mode of CIED was ICD (DDD or VVI) by 11 (39.28%) and DDDR by 33 (35.4%) for the EG and woEG, respectively. The most frequently used type of leads was bipolar leads in 19 (67.9%) of patients in EG and in 63 (67.7%) of patients in woEG. The mean utility time of the devices in EG was 1.36±0.5 months whereas it was 55.21±46.8 months in woEG (Table 2).

Responses of patients with CIEDs about physical activities or daily life activities were shown in Table 3. Percent of correct responses to all of the questions asked in this section were significantly increased in patients of EG (p<0.05). Percent of correct answers were significantly different for “using the arm on the side of CIED” and “doing exercise or sports” in patients of woEG compared to EG (p<0.05). Other responses to the question of this section did not differ significantly between groups (p>0.05).

Responses of the patients to questions on safety of household appliances for electromagnetic interference with CIEDs were given in Table 4. Percent of all of the questions correctly answered in this section significantly increased in EG (p<0.05). Furthermore, percent of correct responses in EG significantly exceeded that of woEG on issues of safety on “TV, radio, remote controls”, “electrical lamps, switch buttons”, “microwave ovens”, and “cellular phones”.

Responses of the patients on harmful devices inducing EMI with CIEDs were presented in Table 5. All of the items correctly responded to improved significantly in the EG. Percent of responses related to “power stations, electric generators”, “chainsaws, welding equipment”, and “loudspeakers with magnets” were significantly higher in EG compared to woEG.

Responses of patients on safety of medical devices for EMI with CIEDs were shown in Table 6. Percent of responses did not differ significantly between EG and woEG. Significant improvements in percent of correct responses were seen on “magnetic resonance imaging (MRI)”, “ultrasonography”, “computed tomography”, and “electrocardiographs” in EG (p<0.05).

## 4. Discussion

Our results indicated that a constructed patient education interview within the context of phase I cardiac rehabilitation significantly improved awareness of patients who were the first time users of the device. This study indicated differences on topics related to physical and daily life activities, doing exercise or sports, using the arm on the side of CIEDs and on EMI of household items, harmful equipment, and some of the medical devices compared to the case before interview. Moreover, the percent of correct responses were found to be significantly higher for EMI of household appliances, medical devices, and all of the harmful equipment compared to woEG.

Patients might encounter new problems after discharge at home and develop concerns and uncertainty about living with CIEDs [6, 7]. It was observed that lack of information or misinformation may result in self-imposed restrictions that can adversely affect ordinary activities and create hesitancy about electrical items found in the house setting [20, 21]. It was demonstrated that the most frequent question directed to healthcare providers was related to the motion and effort that was followed by environmental influences [9–11, 22]. In fact, both PMs and ICDs have a risk of device malfunction related to external factors leading to morbidity, mortality, or device replacements; moreover ICDs have a risk of giving inappropriate shocks [23].

### 4.1. Daily Life and Physical Activities

A study from South Africa showed that up to 50% of the patients felt handicapped after the device implantation and 53% of the patients felt they were less active after the device [24]. Another study from Pakistan found that considerable proportion of patients with PMs think that they should not perform many daily routine activities including climbing stairs, bending over during praying, or sleeping on the side of the PM [20].In this study, patient education interview significantly increased the awareness of patients in EG group in many aspects of daily life including “returning to daily life, driving, climbing the stairs, using the arm on the side of CIED, doing exercise or sports, swimming, returning to sexual life and work, bending forward with the trunk”.

The percent of correct answers were similar to that of patients in woEG for all questions in this section except for “using the arm on the side of CIED and doing exercise and sports”. Patients in EG were requested to limit the excessive range of motion in the shoulder on the side of CIED to prevent lead displacements during the healing period. Additionally, they were informed about restrictions on weight lifting and repetitive use of that arm. Therefore, correct responses might be reduced in patients in EG who were in the healing period and would soon regain range of motion in the shoulder joint. Actually, patients in woEG who had been living with the device for 55.21±46.8 months probably might have learned that daily activities could be done safely by experience rather than knowledge. This fact was reflected in the percent of correct responses in woEG group ranging from 40.9% to 87.1% in this section. This observation was similar to the findings of Rassin et al., who detected that questions directed to healthcare providers were highly related to daily, routine activities in the first period following implantation which were replaced by nondaily activities and situations later on [21]. Therefore, it can be considered that patients in EG could achieve a perception of safety about daily activities much earlier than patients in woEG.

The extent to which the physical activities can be permitted is determined by both primary arrhythmic indication and the cardiopulmonary capacity [17]. Current guidelines for ICD patients in both Europe and the United States recommend against any competitive sports more vigorous than class IA activities, including bowling or golf [25]. The recommendation for patients with PMs is avoidance of sports with danger of bodily collision [26]. Patients in EG were informed that they could do exercise or be involved in sports activity unless restricted by the cardiologist. A higher percent of patients correctly responded to this item in EG which caused a significant difference compared to woEG.

78.6% and 61.3% of the patients in EG and woEG considered sexual life as safe. Furthermore, the doubt related to sexual life improved significantly within EG. This ratio was close to these found in the study of Cutitta in which 64.6 % of patients with ICD reported ability to engage in sexual activity, in contrast to 51.0% who avoided sexual activity [27]. Fear of shock or increase in heart rate, doctor instruction, and lack of desire were reported as reasons for avoidance [27, 28].

Driving is restricted for patients with ICDs, as long as 6 months according to Canadian Cardiovascular Society [29] and 3 months according to European guidelines [30] for secondary prevention. 1 week of restriction is recommended to allow healing following PM implantation or generator replacement of either PM or ICD [26]. It is evident that patients in EG and woEG did not hesitate about driving. The higher percent in woEG group might be related to the completion of the restriction period although difference was insignificant between groups.

Returning to work is presumably affected by the underlying cardiac disease and by the implanted device in patients with PM and ICD. Except industrial jobs the risk of EMI in the work place is low and working place analysis including noise field measurement can be made under suspicion [17]. Patients in the EG significantly developed perception of safety about returning to work which was indifferent from that of patients in woEG who had returned to ordinary life for a long time.

### 4.2. EMI with Household Appliances

Advancements in both the number of patients with CIEDs and the technology that emits electromagnetic signals and that can potentially interfere with CIED function made us to consider EMI with devices found in home, medical, and work environments. Patients can minimize EMI by maintaining distance as large as possible between the CIED and the EMI source and decreasing the time of exposure. On the other hand, they cannot control some of the factors such as intensity of the electromagnetic field and signal frequency [31].

Percent of correct responses ranged from 35.7% to 82.1% after intervention and were related to safety of frequently used household appliances in EG. Interestingly percent of correct answers related to very basic household appliances such as “TV, Radio, remote controls”, “electrical lamps, switch buttons”, “microwave ovens”, and “cellular phones” were significantly lower in woEG. Considering that the instructions related to each of the devices were provided during the interview, it can be speculated that patients in EG tended to learn and remember the information related to most frequently used items in their life. However, long term users were unaware of much of the basic safety information related to CIEDs.

Highest incidence of interference with cellular phones was noticed when they were placed directly over the CIED itself [32] or positioned* <*10 cm from the CIED during the ringing phase [32–34]. Generally, physicians advice using the contralateral ear to the CIED and avoiding physical proximity [33–35]. The percent of correct answers increased significantly in EG group for cellular phones. This significant difference also was present compared to woEG indicating that interview could decrease unnecessary fears of patients towards cellular phones.

### 4.3. EMI with Harmful Equipment

Awareness against equipment with harmful EMI [19, 31, 32] such as “power stations, electric generators”, “chainsaws, welding equipment”, “base stations, cell sites”, “amateur/ham radio stations, radiofrequency transmitters, transformer”, “ignition system of automobiles or motor cycles”, and “loudspeakers with magnets” increased significantly compared to our knowledge before intervention. EG provided a significantly higher percent of correct information compared to woEG related to some of the harmful equipment. The harmful effects of base stations and cell sites that were necessary for widely used mobile phones were well known by the patients in both groups which were reflected as nonsignificant difference.

### 4.4. EMI with Medical Devices

EMI device interactions are most likely to happen in the medical environment. The need for the procedure or test, the dependency status of the patient, and optimal device programming must be assessed to decrease EMI and device malfunction [31].

Patients in EG increased their awareness about safety of “MRI, ultrasonography, computed tomography, electrocardiographs” significantly; however, there was no significant increase for “X-ray, lithotripsy, radiotherapy, electrical devices of dentist (such as dental drills), electrocautery”. Once more, we can speculate that patients might have increased the knowledge related to medical devices they commonly encounter during medical investigation. It was not possible to calculate significance for “TENS (transcutaneous electrical nerve stimulation)” and “short wave radiofrequency (diathermy)” due to the missing number of responses.

Limited health literacy associated with education, ethnicity, and age creates a handicap for adaptation to living with CIEDs [13]. A great proportion of our patients had low or moderate education level in our study which necessitated simplification of the language because the quality of care highly depends on communication between the physician and the patient so that appropriate information is exchanged. Furthermore, social environment of the patients might be a source of misinformation and misperceptions. Educational interventions must provide current information regarding the treatment and underline behavioral strategies to avoid complications and live safely with them [36]. Healthcare professionals should provide sufficient time, education, and supportive communication to increase acceptance of the device [37]. Malm et al. pointed out the importance of providing clear, relevant information, based on the patient's needs, while the patients are still in the hospital enabling patients to manage their life situations better in the recovery phase and have better quality of life later on [10]. Therefore, a patient based educational interview might be superior to other sources of information such as booklets or Internet. Constructed support from healthcare professionals was found to contribute to increased acceptance of the device in the study of Ingvild et al. [37]. Lewin et al. showed that a brief cognitive behavioral rehabilitation programme for patients with ICDs was cost effective due to improved health-related quality of life, psychological endpoints, and reduced number of unplanned admissions [15]. Another study in patients with ICDs showed that a targeted patient-centered educational video could improve knowledge of sudden cardiac arrest and reduce racial differences in ICD preferences [38]. Psychoeducational interventions made even with telephone counseling were found to reduce psychological symptoms and decrease disability days or calls to providers and improve knowledge about sudden cardiac arrest in patients with ICDs [14, 39]. However, the impact of patient education intervention on the awareness of living with CIEDs has never been studied in the literature although education with varying contents was provided as a part of psychoeducational program in limited number of studies. The effect of the education was demonstrated with respect to preintervention in EG. Furthermore, postintervention ratios were compared with woEG that cannot be considered as a pure control group as they have been living with devices and gathered information without education program from sources such as medical personnel, mass media, and social sources.

The effect of education was evaluated by items in the survey with published correct answers in NASPE guidelines [19]. Therefore, the right versus wrong nature of the survey did not necessitate reliability measurement; however internal consistency of the survey was high. As a limitation, the patients included in this study were collected from a territory reference hospital and the study reflects regional rather than national results. Widespread phase I cardiac rehabilitation programs for patients with CIEDs are necessary to understand the effect of these programs.

## 5. Conclusion

This study showed that a constructed patient education intervention on living with CIEDs within the context of phase I cardiac rehabilitation significantly improved awareness of patients on several aspects of life and EMI. Patients with education responded better than the patients who had been living with devices for some time in some of the questions. Therefore, we can propose that patients with educational interview might have achieved a faster adaptation to daily life and decrease misinformation and misperceptions and also promote the quality of life after the device implantation. Furthermore, the education would help them to be more cautious against environmental factors generating EMI which might subsequently reduce device malfunction.

## Figures and Tables

**Table 1:** Demographic characteristics of the patients with cardiac implantable electronic devices.

**Demographic Characteristics**	**Patients with education** Number (%) (n:28)	**Patients without education** Number (%) (n:93)
Age (years) (mean±SD)	60.54±10.58	65.41±14.40
Sex (M/F)	17/11 (60.7/39.3)	59/34 (63.4/36.6)

Education		
Illiterate	6 (21.4)	17 (18.27)
Primary & Secondary School	16 (57.1)	52 (55.91)
High School	6 (21.4)	13 (14)
University	0 (0)	1 (1.1)

Occupation		
Retarded	14 (50)	48 (51.61)
House Wife	10 (35.7)	34 (36.55)
Self-employed	3 (10.7)	8 (8.60)
Civil servant & student	1 (4.6)	3 (3.22)

**Table 2:** Diagnosis, underlying etiology, type/mode and lifetime of the cardiac implantable electronic device (CIED), and generator replacement number of the patients.

	**Patients with education** Number (%) (n:28)	**Patients without education** Number (%) (n:93)
Diagnosis		
Sinus Node Dysfunction	8 (28.5)	31 (33.3)
Atrioventricular Node Dysfunction	11 (39.2)	37 (39.8)
Ventricular Tachycardia/Fibrillation	6 (21.4)	10 (10.7 )
Severe Heart Failure	3 (10.7)	15 (16.2)

Etiology		
Ischemic Heart Disease	6 (21.4)	30 (32.2)
Post-Myocardial Infarction	1 (3.6)	5 (5.3)
Cardiomyopathy	13 (46.4)	11 (11.8)
Degenerative	2 (7.1)	28 (30.1)
Iatrogenic	-	3 (3.2)
Idiopathic	6 (21.4)	16 (17.2)

CIED type or mode*∗*		
VVI	1 (3.6)	12 (12.9)
VDDR	5 (17.9)	20 (21.5)
DDDR	3 (10.7)	33 (35.4)
ICD (DDD or VVI)	11 (39.28)	18 (19.4)
ICD-CRT	8 (28.6)	10 (10.8)

Polarity		
Unipolar	5 (17.9)	30 (32.6)
Bipolar	19 (67.9)	63 (67.7)

Utility time of current device (months)	1.36±0.5	55.21±46.8

*∗*According to NASPE/BPEG Generic Pacemaker Code (NBG), 2002.

ICD: implantable cardioverter-defibrillators.

ICD-CRT: implantable cardioverter-defibrillators and cardiac resynchronization therapy.

**Table 3:** Correct responses of patients with cardiac implantable electronic devices (CIED) for physical or daily life activities.

**Could patients with CIED's.....?**	**Education Group**		**Group without education** **(n:93)**	**Within education group** **p**	**Between groups** **p**
	**Before education** **(n:28)**	**After education** **(n:28)**			
return to their daily life	13 (46.4)	22 (78.6)	81 (87.1)	**0.012** *∗*	0.266
drive	6 (21.4)	16 (57.1)	65 (69.9)	**0.013** *∗*	0.209
climb the stairs	11 (39.3)	26 (92.9)	78 (83.9)	**0.001** *∗*	0.230
use their arm on the side of CIED	5 (18.5)	16 (57.1)	72 (77.4)	**0.006** *∗*	**0.035** ^**#**^
do exercise or do sports	10 (35.7)	23 (82.1)	55 (59.1)	**0.002** *∗*	**0.026** ^**#**^
swim	2 (7.1)	11 (39.3)	38 (40.9)	**0.012** *∗*	0.882
return to their sexual life	7 (25)	22 (78.6)	57 (61.3)	**0.001** *∗*	0.092
return to their work	8 (28.6)	21 (75)	71 (76.3)	**0.001** *∗*	0.884
bend forward with the trunk	2 (7.1)	17 (60.7)	72 (77.4)	**0.001** *∗*	0.079

*∗*p<0.05, for the comparison within the education group.

**#**p<0.05, for the comparison between groups after education and without education.

**Table 4:** Correct responses to questions on safety of household appliances for electromagnetic interference with cardiac implantable electronic devices.

**Is it safe to use....?**	**Education Group**		**Group without education** **(n:93)**	**Within education group** **p**	**Between groups** **p**
	**Before education** **(n:28)**	**After education** **(n:28)**			
hair dryers, electrical shavers, electrical knife	4 (14.3)	12 (42.9)	23 (24.7)	**0.039** **∗**	0.064
TV, Radio, remote controls	3 (10.7)	22 (78.6)	49 (52.7)	**0.001** **∗**	**0.015** ^**#**^
Computers, CD/DVD or music players	3 (10.7)	17 (60.7)	44 (47.3)	**0.001** **∗**	0.214
electrical lamps, switch buttons	6 (21.4)	23 (82.1)	46 (49.5)	**0.001** **∗**	**0.002** ^**#**^
microwave ovens	1 (3.6)	11 (39.3)	18 (19.4)	**0.006** **∗**	**0.030** ^**#**^
magnetic pads, pillows, mattress	3 (10.7)	12 (42.9)	42 (45.2)	**0.012** **∗**	0.830
electrical muscles stimulators (such as ab stimulator)	6 (21.4)	14 (50)	41 (44.1)	**0.039** **∗**	0.582
treadmills or electrical powered bicycles	2 (7.1)	10 (35.7)	21 (22.6)	**0.021** **∗**	0.163
cellular phones	6 (21.4)	16 (57.1)	28 (30.1)	**0.021** **∗**	**0.009** ^**#**^
passing through security gates in airports	28 (100)	2 (7.1)	12 (12.9)	-	0.403

*∗*p<0.05, for the comparison within the education group.

**#**p<0.05, for the comparison between groups after education and without education.

**Table 5:** Correct responses to questions on harmful equipment for electromagnetic interference with cardiac implantable electronic devices.

**Is it safe to be close to or use....?**	**Education Group**		**Group without education** **(n:93)**	**Within education group** **p**	**Between groups** **p**
	**Before education** **(n:28)**	**After education** **(n:28)**			
power stations, electric generators	9 (32.1)	23 (82.1)	50 (53.8)	**0.001** **∗**	**0.007** ^**#**^
chainsaws, welding equipment	7 (25)	20 (71.4)	44 (47.3)	**0.001** **∗**	**0.025** ^**#**^
base stations, cell sites	6 (21.4)	21 (75)	58 (62.4)	**0.001** **∗**	0.218
amateur/ham radio stations, radiofrequency transmitters, transformers	8 (28.6)	17 (60.7)	53 (57)	**0.035** **∗**	0.726
ignition system of automobiles or motor cycles	7 (25)	17 (60.7)	37 (39.8)	**0.013** **∗**	0.051
loudspeakers with magnets	7 (25)	25 (89.3)	46 (49.5)	**0.001** **∗**	**0.001** ^**#**^

*∗*p<0.05, for the comparison within the education group.

**#**p<0.05, for the comparison between groups after education and without education.

**Table 6:** Correct responses of patients on safety of medical devices for electromagnetic interference with cardiac implantable electronic devices.

**Is it safe to undergo investigation with or treated with....?**	**Education Group**		**Group without education** **(n:93)**	**Within education group** **p**	**Between groups** **p**
	**Before education** **(n:28)**	**After education** **(n:28)**			
magnetic resonance imaging	7 (25)	20 (71.4)	55 (59.1)	**0.001** **∗**	0.240
x-ray	6 (21.4)	11 (39.3)	55 (59.1)	0.180	0.064
ultrasonography	6 (21.4)	18 (64.3)	53 (57)	**0.004** **∗**	0.492
computed tomography	4 (14.3)	13 (46.4)	33 (35.5)	**0.012** **∗**	0.296
electrocardiographs	5 (17.9)	22 (78.6)	68 (73.1)	**0.001** **∗**	0.562
TENS (transcutaneous electrical nerve stimulation)	0 (0)	2 (7.1)	14 (15.1)	-	0.279
lithotripsy	2 (7.1)	8 (28.6)	22 (23.7)	0.070	0.597
radiotherapy	2 (7.1)	8 (28.6)	22 (23.7)	0.070	0.597
short wave radiofrequency (diathermy)	0 (0)	3 (10.7)	11 (11.8)	-	0.858
electrical devices of dentist (such as dental drills)	8 (28.6)	12 (42.9)	53 (57)	0.344	0.189
electrocautery	2 (7.1)	6 (21.4)	14 (15.2)	0.289	0.440

*∗*p<0.05, for the comparison within the education group.

#p<0.05, for the comparison between groups after education and without education.

## Data Availability

The data used to support the findings of this study are available from the corresponding author upon request.
